# Morphology of Nasonov and Tergal Glands in *Apis mellifera* Rebels

**DOI:** 10.3390/insects13050401

**Published:** 2022-04-22

**Authors:** Aneta Strachecka, Jacek Chobotow, Karolina Kuszewska, Krzysztof Olszewski, Patrycja Skowronek, Maciej Bryś, Jerzy Paleolog, Michał Woyciechowski

**Affiliations:** 1 Department of Invertebrate Ecophysiology and Experimental Biology, University of Life Sciences in Lublin, Doświadczalna 50a, 20-280 Lublin, Poland; patrycja.skowronek@up.lublin.pl (P.S.); maciej.brys@up.lublin.pl (M.B.); jerzy.paleolog@up.lublin.pl (J.P.); 2 Faculty of Biology and Biotechnology, Maria Curie-Sklodowska University, Akademicka 19, 20-400 Lublin, Poland; jacek.chobotow@poczta.umcs.lublin.pl; 3 Institute of Environmental Sciences, Jagiellonian University, Gronostajowa 7, 30-387 Krakow, Poland; k.kuszewska@uj.edu.pl (K.K.); michal.woyciechowski@uj.edu.pl (M.W.); 4 Faculty of Animal Sciences and Bioeconomy, Institute of Biological Basis of Animal Production, University of Life Sciences in Lublin, Akademicka 13, 20-950 Lublin, Poland; krzysztof.olszewski@up.lublin.pl

**Keywords:** *Apis mellifera*, cells, Nasonov gland, pheromones, queen, rebels, tergal gland, workers

## Abstract

**Simple Summary:**

Communication in a colony of social insects, such as the honeybee, is possible thanks to the pheromones secreted by all individuals. Pheromones are produced and secreted by the glands. Examples of such structures are Nasonov and tergal glands. Nasonov glands are characteristic of worker bees, while tergal glands are primarily found in queens. There are situations in the colony in which the queen and her pheromones are missing. In these instances, the larvae develop into rebels, which are reproductive workers. We therefore assumed that the rebels would have a reduced Nasonov gland and developed tergal glands. Our assumption turned out to be correct. These discoveries bring us closer to explaining the evolutionary formation of different castes of honeybees.

**Abstract:**

Social insect societies are characterized by a high level of organization. This is made possible through a remarkably complex array of pheromonal signals produced by all members of the colony. The queen’s pheromones signal the presence of a fertile female and induce daughter workers to remain sterile. However, the lack of the queen mandibular pheromone leads to the emergence of rebels, i.e., workers with increased reproductive potential. We suggested that the rebels would have developed tergal glands and reduced Nasonov glands, much like the queen but contrary to normal workers. Our guess turned out to be correct and may suggest that the rebels are more queen-like than previously thought. The tergal gland cells found in the rebels were numerous but they did not adhere as closely to one another as they did in queens. In the rebels, the number of Nasonov gland cells was very limited (from 38 to 53) and there were fat body trophocytes between the glandular cells. The diameters of the Nasonov gland cell nuclei were smaller in the rebels than in the normal workers. These results are important for understanding the formation of the different castes of *Apis mellifera* females, as well as the division of labor in social insect societies.

## 1. Introduction

Social insect societies are characterized by a high level of organization. This is exemplified by the division of labor in reproductive bees and worker bees based on their life expectancies. This order is mediated through a remarkably complex array of pheromonal signals produced by all members of the colony and regulated by social contexts [1,2]. Pheromone signals in honeybees are often enhanced by synergy and the context in which they are deployed and mediated through both temporal and spatial distribution [3,4,5,6]. Nowadays, around 50 chemical substances are known to be essential to the functioning of the society [3,7].

Evolutionary changes in chemical production have been instrumental to the emergence of interactions both within and between species, with behaviors as diverse as chemical defense, pheromonal communication and parental care relying on the transmission of information or resources embedded in chemical secretions [8]. Queen pheromones, which signal the presence of a fertile female and induce daughter workers to remain sterile, are considered to play a key role in regulating the reproductive division of labor in insect societies. Although queen pheromones were long thought to be highly taxon-specific, recent studies have shown that structurally related long-chain hydrocarbons act as conserved queen signals across several independently evolved lineages of social insects. These results imply that social insect queen pheromones are ancient and are likely derived from an ancestral signaling system that was present in their common solitary ancestors [9]. It is unclear whether this conservative character only applies to the compounds that comprise pheromones, or if it also applies to the morphology of the cells in which pheromones are produced and secreted. Raguso et al. [10], Tittiger [11], and Brückner and Parker [8] emphasized that knowledge of cytology, morphology and molecular mechanisms, as well as an understanding of the chemical release mechanisms of cells (the identities of molecular components that regulate subcellular exchange and secretion of chemical signals) is absent for the majority of gland cell types. Our research may help to fill this gap in our knowledge.

In a honeybee *Apis mellifera* colony, the secretion of one pheromone stimulates the reaction and secretion of another in individuals of the same or another caste. The pheromones of mandibular (QMP) and tergal glands in queens, as well as the secretion of workers’ Nasonov glands, are an example of such caste actions [12,13]. Tergal gland (Renner and Baumann glands, located on tergites II–IV) pheromones support QMP functions [14,15,16]. Secretions from the queen’s mandibular and tergal glands evoke the retinue behavior of workers, as well as the effect of ovarian development inhibition in workers [17,18,19]. Moreover, the secretions from these three glands have a cohesive effect in instances of swarm clustering [20,21]. After swarming, when the old queen leaves the nest accompanied by a group of workers to establish a new colony, the remaining workers in the old nest care for the eggs, the larvae of younger workers and developing sister queens [22]. Woyciechowski et al. [23] suggested that information regarding the absence of the queen and her pheromones is transmitted via trophallaxis to worker larvae, which can then change their developmental strategy. As a result, rebels develop from the worker larvae. In contrast to the normal sterile workers, the rebels are primed to reproduce rather than participate in the rearing of the next generation of sister-queen offspring [24]. They have more ovarioles in their ovaries, as well as better developed mandibular glands and underdeveloped hypopharyngeal glands. Moreover, their ovaries are activated regardless of whether they live in queen-less or queen-right colonies [24,25,26,27]. Since the rebels are so anatomically and behaviorally different from the normal workers and more queen-like, the following questions arise: How do their gland cells function, and are they morphologically similar to the gland cells of queens or workers? Since the tergite glands are characteristic of *A. mellifera* queens and the Nasonov glands of workers, disturbances in these systems lead to an imbalance in reproductive dominance in the colony [28,29,30]. To answer these questions, we dissected cells from the tergal glands of rebels and compared them with those of queens and normal workers. We also dissected cells from the Nasonov glands of rebels and compared them with those of normal workers.

## 2. Materials and Methods

This study was performed at the apiary of the University of Life Sciences in Lublin, Poland (51.224039 N–22.634649 E). We used four colonies of *A. m. carnica* honeybees; three of them—the source colonies—were used to obtain larvae of known ages to rear normal workers and rebels and one (colony 4) was used for rearing queens.

### 2.1. Experimental Design

The queens were taken from each of the three unrelated source colonies, each of which populated two-box hives (Dadant Blatt; 20 frames; 435 × 150 mm^2^). They were caged within a queen-excluder comb-cage containing two empty combs (C1 and C2) for 24 h, with the purpose of laying eggs. On the third day after the end of egg laying, 50 one-day-old (12–24-h-old) larvae from C1 and C2 were grafted into queen cell cups suspended vertically in the colony (No. 4, according to Büchler et al.’s [31] method). After the larvae were grafted, C1 and C2 were restored to their source colonies with the remaining larvae. Next, each of the source colonies was divided into two equal parts with each in a separate box, according to Woyciechowski and Kuszewska’s [24] procedure. The first part (top box), containing the queen, workers, brood and C1, was used for rearing normal (non-rebel) workers, whereas the other part (bottom box), without a queen but with workers, brood and C2, served for rearing rebels. After sealing the larval cells in C1 and C2, the two boxes were put together again, respectively, to restore each of the three source colonies. After 15 days from the moment the eggs were laid, sealed queen cells were placed in an incubator (temperature 34.5 °C, relative humidity 60%). Soon after, the one-day-old queens were placed in mini-hives with about 200 nursing workers. The seven-day-old queens were used for the morphological analyses. After 18 days, brood combs C1 and C2 were also placed in the incubator. Freshly emerged rebels and normal workers, marked with different colors on the thorax, returned to their colonies. For the morphological analyses, 20 seven-day-old rebels and 20 seven-day-old normal workers were captured from each of the three source colonies.

### 2.2. Morphological Analyses of the Gland Cells

The Nasonov glands were dissected (Stereo Zoom Microscope Olympus SZX16; Camera: Olympus DP72; Warsaw, Poland) from each of the 60 rebels and 60 normal workers. The tergal glands from the third, fourth and fifth tergites were dissected from each of the 60 queens, as well as from each of the 60 rebels and 60 normal workers (the same as those used in the Nasonov gland preparation). Each of the glands was placed on glass slides in 0.6% natrium chloratum (pro inj.) and covered with cover-glasses. The gland cells were observed and photographed with an Olympus DP 72 camera (Microscope Olympus BX61; magnification × 40) with a DIC attachment. This method enables the undistorted visualization of living tissues (see [32]). The diameters of the gland cell nuclei were measured using the Olympus software.

### 2.3. Examination of Anatomical Parameters

In order to confirm whether the emerging bees were normal workers or rebels and verify the queen status, Woyciechowski and Kuszewska’s [24] method was used to determine the number of ovarioles (ovarian tubules) in both ovaries.

The highest number of tubes was found in all the dissected queens (199.6 ± 25.4). The normal workers had fewer ovarioles (5.1 ± 1.1) than the rebels (12.4 ± 1.8). Significant differences between these results allowed us to continue our research and compare the glands in three different groups of females.

### 2.4. Statistical Analysis

The results were analyzed using Statistica, version 13.3 (2017) for Windows, StatSoft Inc., USA. The mixed-model two-way ANOVA followed by the post hoc Tukey HSD test were used to compare the number of ovarioles and the diameters of the Nasonov gland cell nuclei between the rebel and normal workers, as well as the queens. The fixed effect was the phenotype of the female (queen, rebel workers, and normal workers). In order to compare the gland-cell nuclei of the tergal gland, the mixed-model three-way ANOVA was used, followed by the post hoc Tukey HSD test. The fixed effect was the phenotype of the female (queen and rebel workers) and the location of the tergal gland (GIII—tergal glands from the third tergites; GIV—tergal glands from the fourth tergites; GV—tergal glands from the fifth tergites).

## 3. Results

### 3.1. The Morphology of the Nasonov Gland

**Figure 1:**
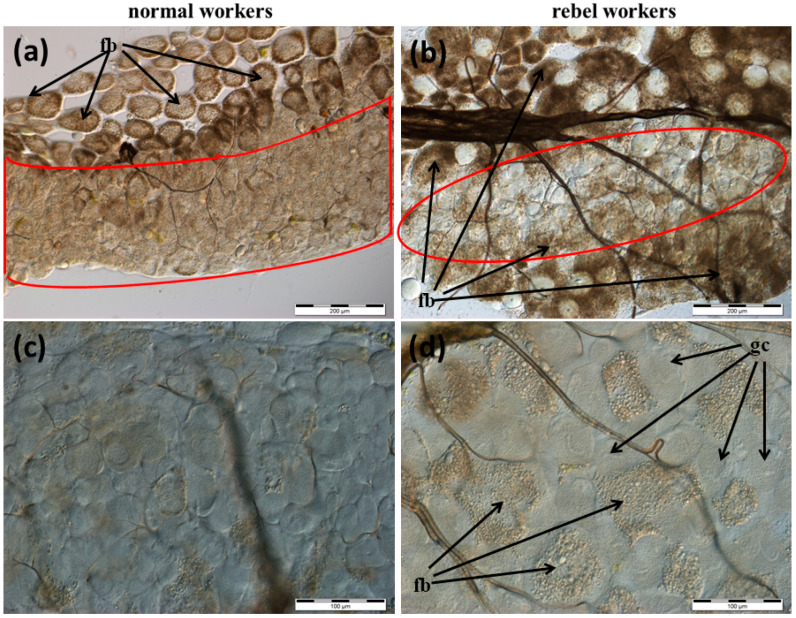
Nasonov glands in normal workers (**a**,**c**) and rebels (**b**,**d**) (gland cells marked in red). Fb—fat body; gc—gland cells.

**Figure 2:**
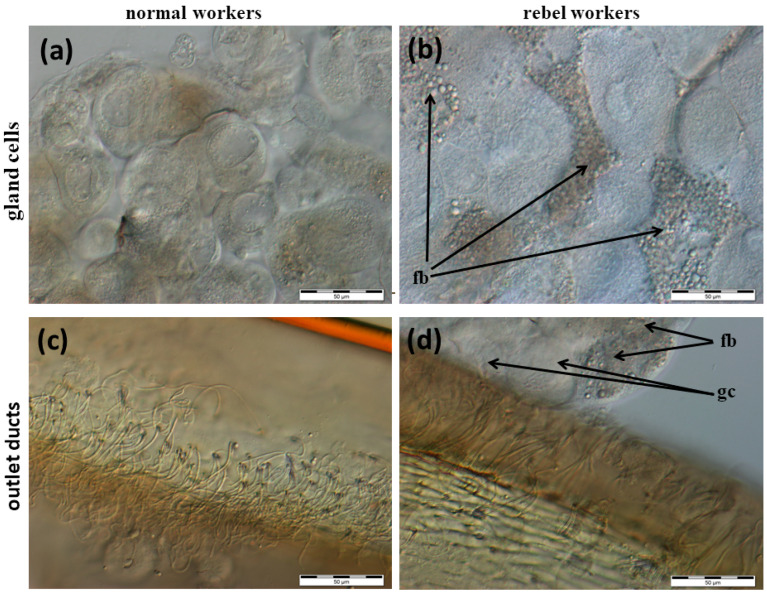
Nasonov glands in normal workers and rebels. (**a**) Gland cells in normal workers; (**b**) gland cells in rebels; (**c**) outlet ducts in normal workers; (**d**) outlet ducts in rebels. fb—fat body cells; gc—gland cells.

**Figure 3:**
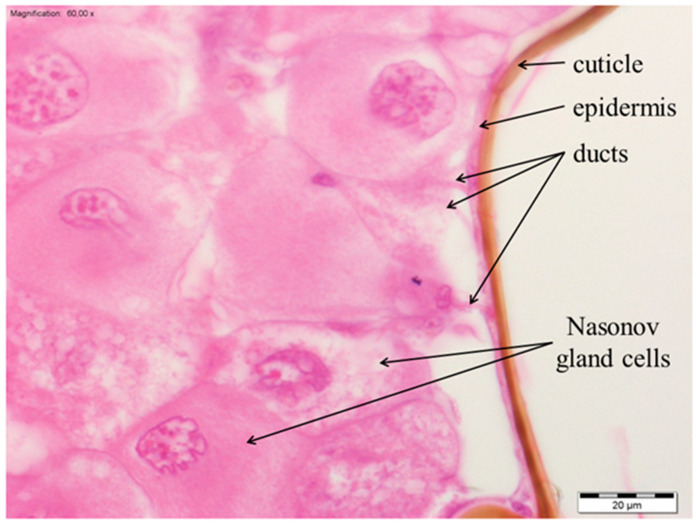
Nasonov gland cells in normal workers. Hematoxylin and eosin staining.

**Figure 4:**
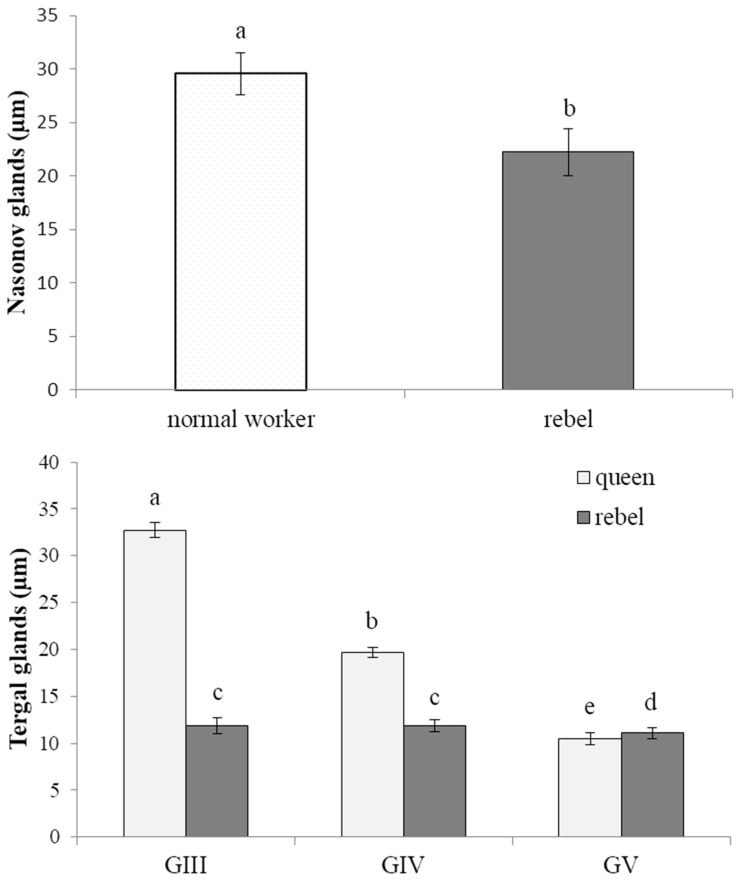
Diameters of the cell nuclei in the Nasonov and tergal glands. The small letters indicate significant differences (*p* ≤ 0.05) between the rebels and normal workers in their Nasonov glands (two-way ANOVA with Tukey multiple comparison colony: F_2,114_ = 0.51, *p* = 0.664; phenotype: F_1,2_ = 248.1, *p* = 0.004; colony * phenotype F_2,114_ = 8.09, *p* < 0.001) and between the rebels and queens in their tergal glands (three-way ANOVA with Tukey multiple comparison: colony: F_2,342_ = 5.2, *p* = 0.006; location of tergal gland: F_2,4_ = 9157.7, *p* < 0.001; phenotype: F_1,2_ = 18,074.3, *p* < 0.001; colony * location of tergal gland: F_4,342_ = 0.1, *p* = 0.995; colony * phenotype: F_2,342_ = 0.7, *p* = 0.517; phenotype * location of tergal gland: F_2,342_ = 8053.3, *p* < 0.001; phenotype * location of tergal gland * colony: F_2,342_ = 3.7, *p* = 0.005). GIII—tergal glands from the third tergites; GIV—tergal glands from the fourth tergites; GV—tergal glands from the fifth tergites.

The Nasonov gland, located just below the intersegmental membrane between the 6th and 7th tergite of the abdomen (Figure S1), forms cells whose exit ducts are located in the duct region (Figure 1, Figure 2 and Figure 3). In normal workers, the package of these cells was stretched to a length of about 1500–2000 µm (Figure 1a); the cells were large with a centrally located nucleus (Figure 1c, Figure 2a and Figure 3). Many cells (from 160 to 277) closely adhered to one another and the ducts departed from each of them (Figure 2c). On the other hand, in the rebels, the number of glandular cells was very limited (from 38 to 53), with a strand length of about 800–1000 µm (Figure 1b). Additionally, there were fat body trophocytes between the glandular cells (Figure 1d and Figure 2b,d). The diameters of the cell nuclei were smaller in the rebels than in the normal workers (Figure 4).

### 3.2. The Morphology of the Tergal Glands

**Figure 5:**
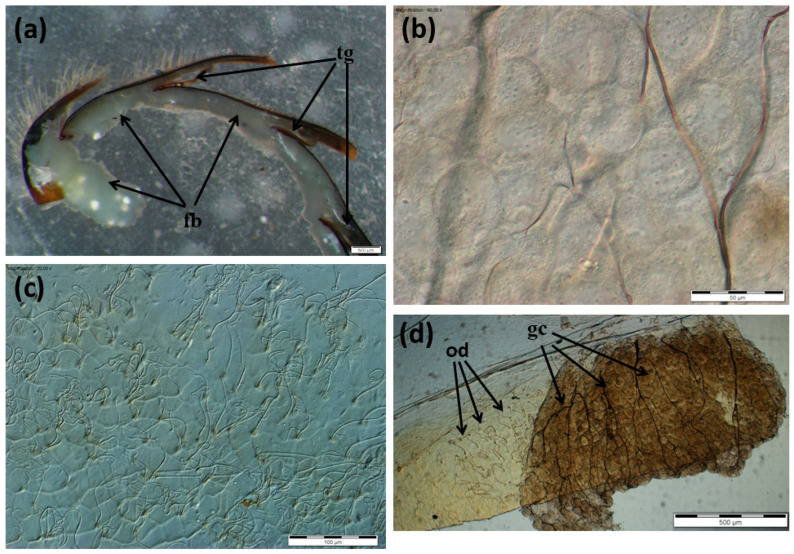
Tergal glands in queens. (**a**) Longitudinal section through the outer layers (cuticle along with the fat body and glands) of a queen abdomen; (**b**) gland cells; (**c**) outlet ducts; (**d**) the intersegmental membrane with the cells of the gland and their outlet ducts. tg—tergal glands; fb—fat body; od—outlet ducts; gc—gland cells.

Packages of tergal gland cells located underneath the abdominal tergites III to V (Figure 5a) were stretched on a length of about 2500–4500 µm in the queens (Figure 5d) and about 1500–3000 µm in the rebels. Normal workers were observed to have 1–3 tergal gland cells, which were very delicate and quickly burst, making it impossible to register their images.

The queens were found to have a lot of cells (there were 25–32 glandular cells on the 200 µm^2^ tissue surface) that closely adhered to one another, with a centrally located nucleus (Figure 5b). From each cell departed the outlet ducts (Figure 5c,d), from which pheromones were emitted with pulsating movements (Video 1). No differences were observed in the morphological images between the glands from various tergites (III, IV and V), but these glandular cells differed in their nucleus diameters—the largest nuclei were found in the third tergite and the smallest ones in the fifth (Figure 4).

**Figure 6:**
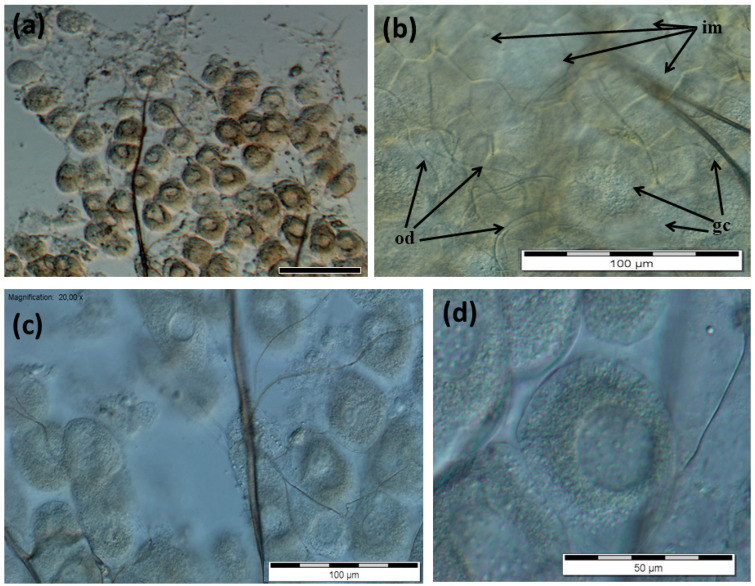
Tergal glands in rebels. (**a**–**d**) Gland cells; od—outlet ducts; gc—gland cells; im—intersegmental membrane.

The glandular cells in the rebels were numerous (there were 15–21 glandular cells on the 200 µm tissue surface) but they did not adhere as closely to one another (Figure 6a–c). Their cell nuclei were centrally located (Figure 6d) and their diameters did not differ between the third and fourth tergites. The diameters of the cell nuclei in the rebels were smaller in comparison with those in the third and fourth tergites of the queens (Figure 4).

## 4. Discussion

The lack of queen pheromones during the larval development of workers has far-reaching effects, not only on their anatomy [23] and behavior [27,33,34,35,36] but also on the morphology of the emerged rebels (Figure 1, Figure 2, Figure 3, Figure 4, Figure 5 and Figure 6). Rebels are focused on their own reproduction [24]. Hence, at the stage of preimaginal development, there must already have been changes in their epigenome [37,38] which lead to the development of tergal glands and the reduction in Nasonov glands (Figure 1, Figure 2, Figure 3, Figure 4, Figure 5 and Figure 6). It can be concluded that the rebels changed their life strategy in order to become as queen-like as possible and achieve personal reproductive success by avoiding worker policing [39].

Billen et al. [15] and Wossler et al. [16] reported that some workers may have tergite glands, but the number of these cells in workers was smaller than in queens. In our experiment, we also observed 1–3 tergal gland cells in normal workers. The morphological structure of these cells was similar to that of the queens. These cells were very fragile, they quickly broke and their measurement and visualization were not possible, contrary to what was observed in the *A. m. carnica* queens (Figure 5). *A. m. scutellata* workers also possessed very few gland cells (mean ± SD; 0.9 ± 0.6), ranging in size from 255 µm^2^ to 1327 µm^2^, whereas *A. m. capensis* workers had on average ten times more cells (9.3 ± 1.7), ranging in size from 723 µm^2^ to 2200 µm^2^ [16]. It can be calculated that the diameters of these cells ranged from 18.02 to 41.11 µm and from 30.34 to 52.93 µm, respectively. In our experiment, we analyzed the diameters of the glandular cell nuclei as a measure of their metabolic activity. This gland assumed considerable sizes in workers with increased reproductive potential, such as rebels (Figure 6), and consisted of numerous active cells, as indicated by the diameter of the cell nuclei (Figure 4) at a mean of 11.61 µm (± 0.78; SD). It is, however, worth emphasizing that the diameters of the nuclei in the rebels did not differ between the third and fourth tergites. The largest nuclei in the queens were observed in the third tergites (32.7 ± 0.8 µm), and the smallest in the fifth tergites (10.5 ± 0.6 µm; Figure 4). This may indicate the functional adaptation and secretory specialization of these cells in queens. Most likely, not all cells in each tergite are simultaneously activated and the cycles of their metabolic activities are probably rotational. Our research shows that the higher the reproductive potential of the female, the greater the specialization and organization of these glands, depending on the segment in which they are located (Figure 4). It is surprising that the rebels had larger cell nuclei in the fifth tergite than the queens. This observation requires further research and explanation. Okosun et al. [40,41] suggested that the workers’ tergal gland secretions included the three ethyl esters (ethyl palmitate, ethyl oleate and ethyl stearate) which have both primer and releaser effects. Due to the presence of esters, the pheromone mixture is attractive for other bees and regulates reproduction, whether it is emitted by the queen [18,42,43] or by workers [41]. The queen-like glandular secretions of reproductively dominant workers allow for the determination of their reproductive dominance [28,41,44]. In queens, this dominance is very strong, due to the gland size as well as the number and types of compounds (long-chain fatty acids, long-chain esters and a series of unsaturated and saturated hydrocarbons as components); [18,43]). Since the number of components in worker pheromones is limited [41], this may explain the lack of differences in nucleus diameters between the third and fourth tergites in the rebels (Figure 4). Thus, tergal gland secretions act synergistically with mandibular gland secretions, which are more developed in the rebels in comparison to normal workers. Moreover, the rebels have underdeveloped hypopharyngeal glands [24], which suggests low production of brood food [45] and restricted nursing activity [46].

In turn, the reduction in the number of Nasonov gland cells, overgrowing with trophocytes and the reduction in the diameter of cell nuclei (Figure 1, Figure 2 and Figure 4) in rebels most likely affects the composition and number of released pheromones. This may suggest a disturbance in the correct orientation of the bees. Thus, intraspecific reproductive parasitism in the rebels is perhaps not only the result of a high reproductive potential and their reproductive strategy, as suggested by Kuszewska et al. [27], but also arises from morphological and functional changes in their Nasonov glands. The limited engagement of the rebels in raising the next generation of bees could result in the reduction in their Nasonov glands (Figure 1 and Figure 2) and pheromone concentrations, as suggested by Al-Kahtani and Bienefeld [21]. Moreover, Bortolotti and Costa [12] stated that the release of the Nasonov pheromone is only stimulated by sugar concentrations. This suggests that the Nasonov pheromone is mainly used to attract workers toward water sources and is involved in nectar source location. This may explain why the rebels display a delayed onset of foraging and a stronger tendency to collect nectar in comparison to normal workers [35]. In other words, by investing in their own egg laying and living a longer life [26] the rebels delay risky tasks, such as foraging [33,47,48,49]. Moreover, dysfunction of the Nasonov glands in rebels can lead to functional disorders related to social immunity (e.g., the removal of dummies) [50]. These mechanisms are controlled by the juvenile hormone and vitellogenin, the concentrations of which in rebels are much higher than in normal workers [51].

All the above-mentioned facts regarding the morphology and anatomy of the rebels are important to clarify the evolutionary strategy of reproduction in workers, which results from the assumption of kin selection theory [52,53] and can also explain the altruistic strategies of colony members [54,55], as well as certain conflicts and behaviors between individuals in a nest [39,56].

Additionally, we wish to draw the reader’s attention to the fact that the main difficulty in analyzing glandular cells, especially those from invertebrates, is their short survival rate. In most studies, such cells are fixed immediately after dissection and only then viewed under a microscope. Our work presents a pioneering approach in imaging glandular cells that can be viewed, measured, and even observed for their release of chemical compounds while maintaining their viability (detailed protocols are described in the Materials and Methods section). By using this method, we have expanded the knowledge of the morphology of the Nasonov and tergal glands in workers with increased reproductive potential, such as the rebels, which in this respect are more like queens than normal workers.

## 5. Conclusions

In order to become as queen-like as possible, rebel honeybees have developed tergal glands and reduced Nasonov glands. Developed tergal glands in rebels, from which pheromones are secreted, are one of the reasons for their temporary reproductive domination in the colony. Moreover, the higher the reproductive potential of the female, the greater the specialization and organization of these glands, depending on the segment in which they are located. The reduction in the number of Nasonov gland cells, overgrowing with trophocytes and the reduction in the diameter of cell nuclei most likely affect the composition and number of released pheromones and suggest a disturbance in the correct orientation of the rebels. Therefore, rebel honeybees are focused on personal reproductive success instead of performing tasks for the colony.

## Data Availability

The datasets generated during and/or analyzed during the current study are available from the corresponding author on reasonable request.

## Supplementary Materials

The following are available online at https://www.mdpi.com/article/10.3390/insects13050401/s1. Figure S1: Nasonov gland in normal workers observed under SEM (according to Ptaszyńska et al.’s [57] method).
