# Supplementary material for: Morphology of Nasonov and Tergal Glands in Apis mellifera Rebels
Source: Insects. 2022 Apr 22;13(5):401. doi: 10.3390/insects13050401 (PMC9146257; doi:10.3390/insects13050401)
Supplement: Supplementary file 1 [file insects-13-00401-s001.zip › insects-1683338-supplementary.pdf]

Supplementary material

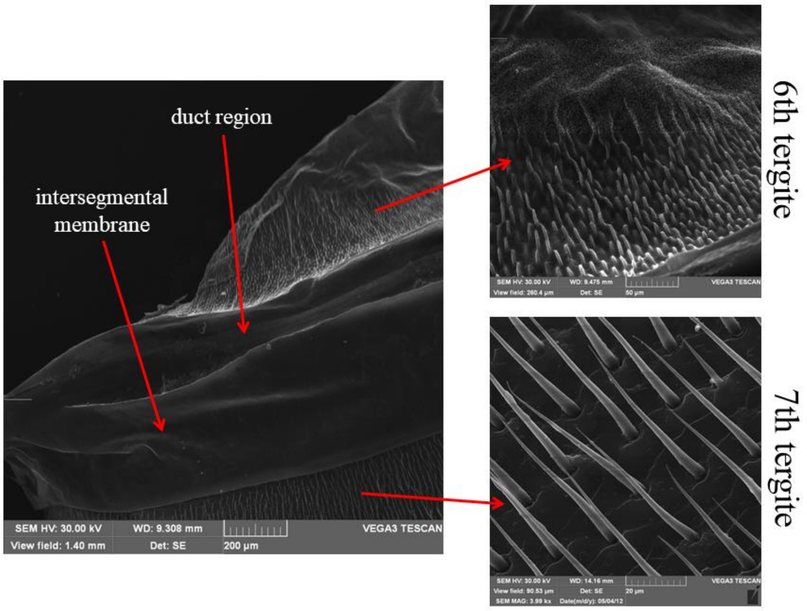

Figure S1. Nasonov gland in normal workers observed under SEM (according to Ptaszyńska et al.'s (2014; DOI: 10.3896/IBRA.1.53.5.02) method).
